# Dworkin’s Paradox

**DOI:** 10.1371/journal.pone.0038529

**Published:** 2012-06-26

**Authors:** Seung Ki Baek, Jung-Kyoo Choi, Beom Jun Kim

**Affiliations:** 1 Integrated Science Laboratory, Umeå University, Umeå, Sweden; 2 School of Economics and Trade, Kyungpook National University, Daegu, Korea; 3 BK21 Physics Research Division and Department of Physics, Sungkyunkwan University, Suwon, Korea; Cinvestav-Merida, Mexico

## Abstract

How to distribute welfare in a society is a key issue in the subject of distributional justice, which is deeply involved with notions of fairness. Following a thought experiment by Dworkin, this work considers a society of individuals with different preferences on the welfare distribution and an official to mediate the coordination among them. Based on a simple assumption that an individual’s welfare is proportional to how her preference is fulfilled by the actual distribution, we show that an egalitarian preference is a strict Nash equilibrium and can be favorable even in certain inhomogeneous situations. These suggest how communication can encourage and secure a notion of fairness.

## Introduction

### Background

The concept of distributive justice has been extensively studied in political philosophy and economics over the past few decades. One of the most important milestones in this field is Rawls’ *A Theory of Justice* [1], which put forward equality as an outcome of the social contract that individuals behind a veil of ignorance should agree on. Even though egalitarian doctrine that all human persons are equal in fundamental worth or moral status is a commonly shared idea, egalitarianism turns out to be a contested concept. There have been several divergent understandings of the meaning of equality, ways to achieve equality, or the metric to measure equality [2], [3]. For instance, someone who puts more emphasis on equality of opportunity may have a very different opinion from those who put emphasis on equality of incomes in spite of the overall agreement on the concept of equality per se.

Among numerous dimensions where egalitarianism varies, the question of what should be equalized is one that many different theories are competing on; is it opportunity, capabilities, resource or welfare [2]–[6]? Dworkin, one of the most influential proponents of resource egalitarianism, admits the immediate appeal of the idea that it must ultimately be equality of welfare insofar as equality is important, and examines the logical consistency and practical applicability of this welfare egalitarianism [7]. According to Dworkin, welfare egalitarianism, concerned with equality in every person’s overall satisfaction, has an inconsistency in its logic. For example, if one accepts the idea that those who are handicapped need more resources to achieve equal welfare, the same argument should apply to those who have expensive tastes for the same reason. However, one should immediately recognize that the appeal of welfare egalitarianism becomes much less strong in the case of expensive tastes than in the case of the handicapped. The fact that the same idea can be accepted in some cases and seems disturbing in other cases reveals a logical inconsistency of welfare egalitarianism. Dworkin also criticized welfare-based egalitarianism on that it inevitably relies on the possibility of interpersonal comparisons of utility, which places a large burden to a policy maker in practice. Lastly, Dworkin argues that it would probably prove impossible to reach a reasonable degree of equality in this conception in a community whose members held very different and very deeply felt political theories about justice in distribution.

The last point is the one that we focus on in this paper. We will show that the existence of contradictory political theories does not immediately lead to the impossibility but can be formulated as dynamics which admits a unique solution under certain assumptions. By doing this, we will argue that the reasoning in [7] can be regarded as a tool to analyze and advocate the idea of equality in welfare. To some extent, this is complementary to a previous work which argues that one can reach the idea of equality in welfare by starting from that of equality in resources [8]. Following the logic that Dworkin used when he showed the impossibility to reach an agreement on redistribution in terms of welfare, we also set aside the issues of logical inconsistency and inter-personal comparisons of individuals’ welfare. To focus on the relationship between individual preferences and resulting welfare, we also set aside the issue of impartiality (see, e.g., [9], [10]). We will further assume that individuals have preferences over the distribution of welfare among them [11], [12]. Many theoretical and experimental studies have shown that people are concerned with equality and fairness and often persist in fairness even when they lose monetary payoffs in doing so, e.g., in the public games, ultimatum games or dictator games. This behavior cannot be explained based on the assumption of self-regarding preferences but of others-regarding preferences or social preferences. Our formulation in this paper can serve as a systematic description for such an approach, because it tells us a way to translate others’ payoffs into an individual’s with respect to individual preference.

### Model

Let the concept of welfare be understood as the fulfillment of preferences [13], including success in political preferences, i.e., opinions of how welfare should be distributed. Then the reasoning by Dworkin [7] argues that such an egalitarian society, where everyone is concerned with the equality, will end up with supporting non-egalitarians by its own logic. Suppose that a bigot enters an egalitarian society, with an opinion that some people deserve more than the others. This person will feel frustrated to see that her political preferences are not accepted by egalitarian neighbors, and her welfare becomes relatively lower than the others’. If there is an official committed to compensating for inequality in welfare, by reallocating resources for example, the bigot should get extra resources from the official due to her political frustration, *because* she does not support the egalitarian idea of the society. This is called Dworkin’s paradox in this work. Particularly we note that it can serve as an idealized model to represent our understanding of a modern democratic society. We will look into this hypothetical society a little closer.

Imagine a society of 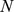 persons and an official. The official, representing a social institution, exists to mediate the global coordination. We assume that the total amount of welfare to be distributed among the persons is fixed as unity, and that the welfare is infinitely divisible, since we are interested only in relative fractions rather than absolute amounts that individuals have. The official herself does not take part in sharing the welfare, but only receives the 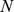 persons’ opinions and find a way to distribute the welfare among them. Let each person 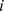 have a certain preference about how the welfare should be distributed, say 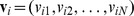 with 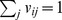. We denote the actual welfare distribution as 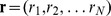. The person 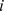
’s welfare is determined by the extent to which her preference is fulfilled. In other words, we consider an equation
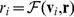
(1)with a certain function 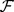, which is assumed to equally apply to all the persons. We suppose that the official wants to announce a stable welfare distribution 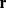 such that each person’s relative share remains unchanged after the announcement, which means that 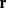 solves Eq. (1) self-consistently. It is important to note that the preferences reported by each individual are assumed to be true and available to the official at every moment, which helps us to focus on basic ideas of the paradox. A few remarks are in order. First, we emphasize that the official plays only a passive role in this setup. As we will see below, the society reaches the same self-consistent solution as long as every person’s welfare becomes public knowledge all the time. The official may guarantee such information to be accessible and accelerate the coordination but the official is basically assigned limited tasks compared to the original argument. Second, related to the first point, we do not require the division of welfare to be impartial from a certain observer’s point of view. Our question is simply how much fulfillment one can get depending on her preference. In this sense, our approach differs from the impartial-division problem [9] and does not touch conceptual difficulties of impartiality (see, e.g., [10]). Finally, individuals are not behind the veil of ignorance. Rather, each of them is supposed to construct a concrete opinion about every other individual using any kind of available information. Although this can impose practical difficulties in a large society, it helps us avoid any theoretical ambiguity or conflict with the ethic of priority [14] found in the veil of ignorance [15].

In order to give a more concrete form to Eq. (1), we first consider how to measure similarity or affinity between distributions and then plug it into Eq. (1). Suppose two arbitrary distributions, 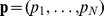 and 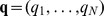, with 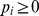, 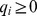, and 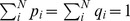. We define a suitable affinity function 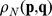 between them, whose specific functional form will be characterized by requiring the following four postulates [16]. First, we postulate separability, which means that one can refine affinity contribution from a certain bin by looking into the bin in a higher resolution without referring to the outside of the bin. Second, we postulate invariance under permutation, because every bin is equivalent. Third, the affinity should be non-negative, i.e., 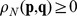, where 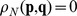 if and only if 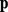 is orthogonal to 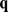, whereas a maximum value is obtained if and only if 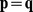. The distributions 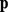 and 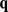 are orthogonal when 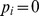 for every non-zero 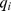 and vice versa. Last, it should be symmetric in the sense that 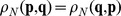, which is intuitively justified. These four postulates characterize our affinity function as
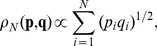
(2)commonly known as the Bhattacharyya measure [17]. While details of the derivation are shown in Supporting Information Text S1, this functional form has a clear geometric interpretation: it can be viewed as a dot product of two vectors 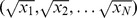 and 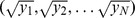, both of which are located on an 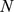-dimensional unit sphere by 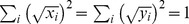. It therefore becomes maximized when two vectors point in the same direction.

It is plausible to assume that the function 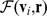 in Eq. (1) will be a non-decreasing function of the affinity between 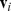 and 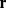, so that 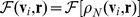. Specifically, we infer that 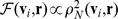, since 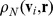 contains dimensionality of 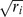 according to Eq. (2). The precise value of the proportionality coefficient should be determined by the normalization condition of 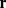. For notational convenience, let us define 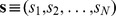 with 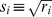 and 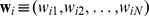 with 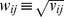. Equation (1) then leads to 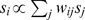, or in a matrix form,
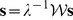
(3)where 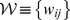 and 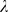 is for normalizing 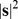, the total welfare. This formalism is reminiscent of the quantum mechanics, where a wavefunction 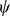 is obtained by solving an eigenvalue problem 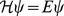 with a Hamiltonian matrix 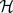 and its eigenvalue 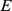. What one can measure in experiments is probability density 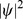. An 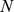-dimensional matrix preserving 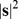 is called orthogonal, and its degrees of freedom is the number of possible planes of rotation in 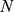 dimension, which is 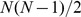. Since 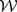 generally has 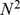 elements and 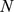 normalization conditions, it has 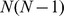 degrees of freedom, so the magnitude of 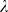 will differ from one in general.

## Results

### Two-person Case

The simplest example of 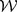 describes a situation where two persons have not ever conceived of each other as a society member to share welfare with. The corresponding matrix is written as.
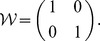



This identity matrix does not change the input state at all, which means that the official cannot really coordinate these two indifferent persons’ opinions in the way that we have assumed. This is actually an example of a reducible matrix [18] or a society that can be divided into smaller pieces: 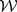 is irreducible if there exists a sequence of 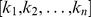 for any 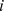 and 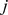 such that 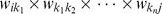 is non-zero. Otherwise, 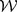 is reducible. Such a reducible case is not our concern since a society is meaningful only when individuals interact with each other. Henceforth, only irreducible cases are considered. Then, unless everyone has zero self-interest, one can prove that there exists a unique stable distribution 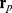 for every 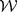 by using the Perron-Frobenius theorem [18]. In other words, 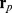 is the only stable fixed point under the action of 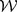, so the official should distribute welfare as given by 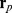.

Let us consider a situation where an egalitarian with 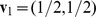 meets a selfish person with 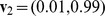. The corresponding matrix formulation will be
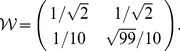
(4)and its stable welfare distribution is obtained by analyzing eigenvectors as 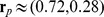. We arrive at this 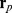 even if we start from 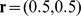 for the following reason: let 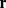 be known to both the persons every time step. The egalitarian first feels happy to see the initial equality in 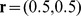, while the selfish person feels unsatisfied, which makes a difference at the next step. The drop in 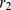 makes the selfish person even more upset, so her welfare continues to decrease until it reaches the stable value, 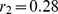.

**Figure 1:**
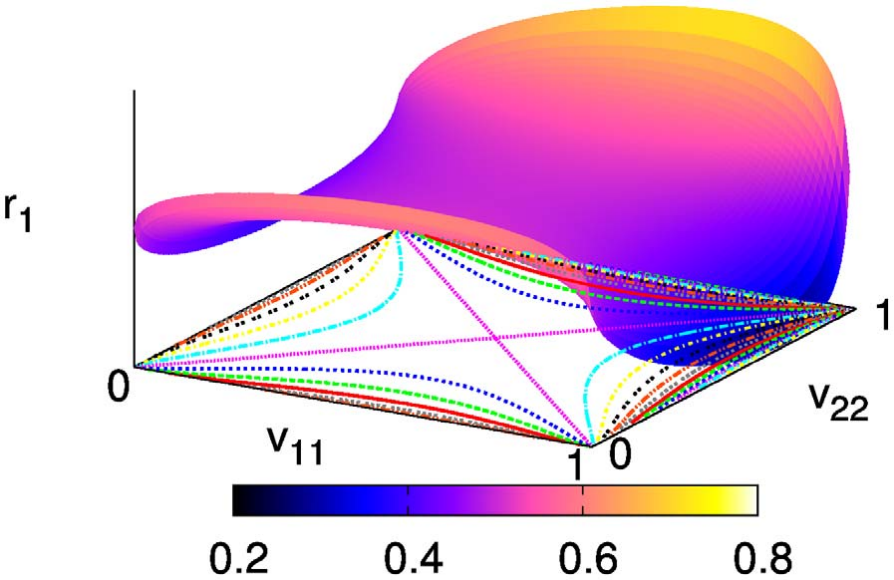
Person 1′s welfare in the two-person case, obtained from Eq. (5). The curves on the plane show contour lines.

We now show that egalitarianism is the minimax solution of this two-person zero-sum game [19]. Generalizing Eq. (4) as
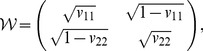
(5)the eigenvalue analysis yields the converged share for the first person, 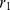, as shown in Fig. 1. It is a saddle-like shape and this person can minimize risk when she has demanded a moderate share of 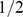 at the first place. The same is true for the other person as well. Although we have assumed fixed preferences in developing the model, if the preferences can evolve in the long run to maximize individual welfare, therefore, this plot shows that this two-person case will lead to an equal welfare distribution.

In practice, a selfish person can be tempted to deceive the official by reporting a false preference to receive a larger share. Provided that person 2 has claimed her self-interest as a certain value 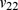, person 1 can always compute the best reply 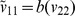 by looking up the maximal share 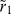 at the given 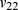 in Fig. 1. Even if her true self-interest 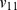 is higher than this false 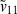, she should still report 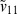 to the official, knowing that she cannot get better than 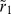 in any way. When person 1 has chosen 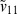 for this reason, the same consideration will lead person 2 to choose 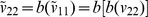, and this reasoning can be repeated between them *ad infinitum*. Such a strategic consideration eventually forces them to choose the egalitarian preference in common, since successive iteration of the best-reply function 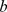 drives every initial input 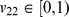 into the egalitarian fixed point, although none of the players are really egalitarians.

### Egalitarianism as a Nash Equilibrium

Let us consider an 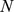-person case where all except one are egalitarian. That is, 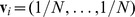 for every 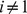. We observe that these 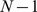 persons will have exactly the same welfare since they always get the same amount of affinity for any welfare distribution 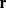. Let us thus denote every egalitarian’s welfare as a single variable 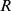. Recalling the separability, we find that all the elements 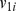 with 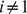 must be the same in order to maximize person 1′s welfare, because her preference about the egalitarians should match with welfare distribution among them. Therefore, person 1 should have a preference of 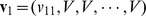 with 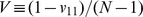. As a consequence, the full 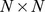 matrix calculation can be simplified to the following 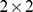 matrix calculation.
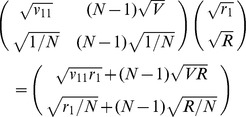
with a normalization condition 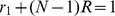. The stable distribution from the simplified matrix then yields 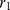 as a function of 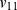, which has 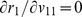 and 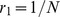 at 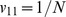. In short, the best possible preference for person 1 is an egalitarian one. If egalitarianism is pervasive, one gets worse off by having another type of preference, which means that egalitarianism is a strict Nash equilibrium [20]. This reproduces Dworkin’s paradox in mathematical terms in the sense that a non-egalitarian in an egalitarian society will have relatively less welfare. A difference from the original paradox is that the official cannot really compensate the non-egalitarian within our formulation since the welfare distribution will converge to the same point again as soon as the compensation is known in public.

### Inhomogeneous Society

Egalitarian preference can be still favorable even when people are all different. For instance, people are not equally born. Let this unavoidable inequality be described by a uniform random variable 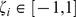. Person 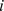
’s overall political preference can be described by another uniform random variable 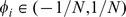: for 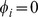, this person is an egalitarian. If 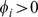, she believes that the better deserve more, while 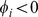 means the opposite. In addition, 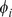 is assumed to be uncorrelated with 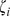. The political preference is then assigned as 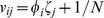, which satisfies 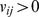. Since 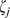 is a relative quantity, one can always subtract an offset value to make 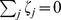, by which the normalization condition 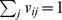 is satisfied. We can obtain the stable distribution by taking 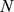 as a very large constant and assuming that 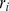 is a function of 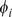 only. By replacing the summation in 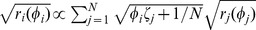 by an integral, we get
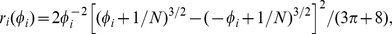
where the proportionality coefficient is determined by the normalization condition. This 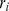 is an even function of 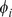 with a maximum 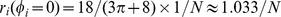, implying the highest fulfillment for an egalitarian. One could point out that this 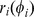 describes just one possible distribution, not necessarily the stable one. However, we see that the integral is positive for all 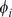, and thus for all 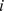, and the Perron-Frobenius theorem tells us that this positivity is true only for the stable distribution [18]. This justifies our starting assumption that person 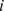
’s welfare 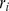 is not determined by her innate part 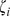 but by her political preference 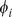 in this society. It is notable that critics have said that the idea of equality in welfare is insensitive to individual responsibility [7]. As explained in [21], one may consider a set of variables characterizing an individual and classify them into two categories: the first category consists of innate properties such as talents that an individual is hardly responsible for. The second category, on the other hand, includes choices and even some of preferences that we can connect to individual responsibility. If we regard 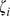 as representing the first category while 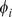 as representing the second category, this example shows that each individual does take responsibility for her political preference but not for her talents. It could be also argued that the limit 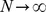 squeezes 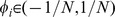 into zero so that the whole problem reduces to the egalitarian society above, where everyone gets 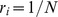. That can be regarded as a first-order approximation of this problem. The calculation given here shows that an egalitarian indeed receives 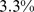 more than in the crude approximation.

### Homogeneously Unequal Preference

In all the cases considered so far, preferences could be said to be neutral on the society level in the sense that there is no systematic bias over the whole society. Let us now imagine that 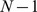 persons with identical preferences, 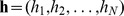, but not necessarily egalitarians. The other person indexed by 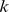 has another type of preference, 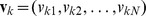. By the similar reasoning as in the egalitarian society, the situation can be simplified to the following 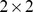 matrix:
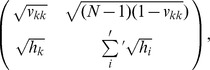
where 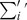 means a summation over 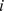 excluding 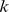. Recall that the 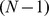 persons with an identical preference have the same amount of fulfillment so person 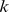 should not distinguish them in order to maximize affinity between her preference and the welfare distribution among them. The above matrix means that we should only determine how to divide welfare between the person 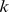 and the other 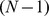 persons. The eigenvalue analysis leads to
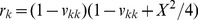
(6)with



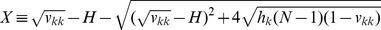
and 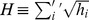. We can differentiate Eq. (6) with respect to 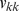 to find the maximum. An easier alternative way is to observe from the separability that the maximum value is obtained when 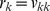, because the question is how to match person 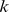
’s preference 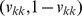 with the welfare distribution 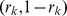, where the second elements represent the whole 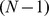 persons. By solving 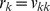 with Eq. (6), one can get 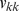 maximizing 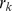 as a solution of the following equation,
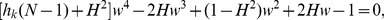
(7)with 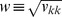. For the egalitarian 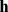 with 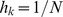 and 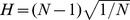, substituting 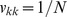 satisfies Eq. (7), consistently with the analysis of the 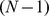 egalitarians. We may also suppose that 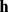 describes an unequal distribution so that the whole society is biased in a certain way. As a specific example, let us assume that 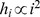, that is, almost everyone wants people with higher indices to have more welfare. With a normalization constant, it should mean that 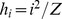 with 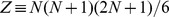, and we thus have



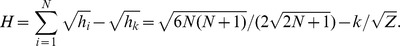



**Figure 2:**
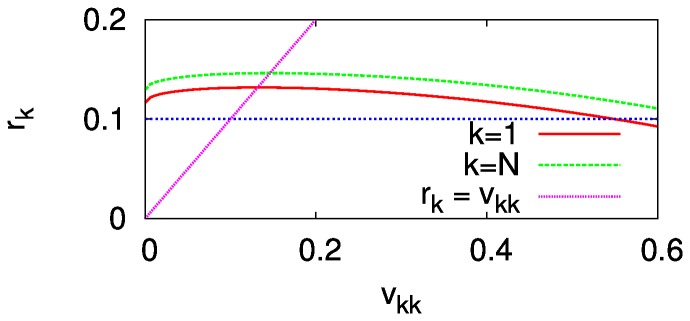
**Equation (6**) as a function of 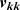 when 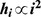 for 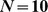. The horizontal line shows 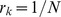. The maximum of 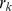 is located at the crossing with the line 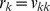.

Inserting this into Eq. (6), we plot 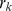 in Fig. 2, where the maximum is found at the crossing with 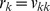. It is a little higher than 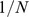 for every 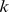. We can do the same calculation for a more severe situation of inequality by setting 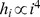, which again yields the same conclusion with a bit larger 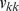. The difference between the optimal 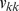 and 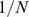 does not vanish as 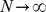 whether 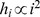 or 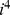. This can be shown by inserting 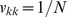 on the left-hand side of Eq. (7) and taking 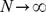, which does not yield zero on the right-hand side. Therefore, the person 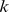 should demand a little more for herself than before and distribute the remainder of the preferences equally to the others, even though they are far from egalitarians, in order to get the maximum welfare.

### Transient Behavior

**Figure 3:**
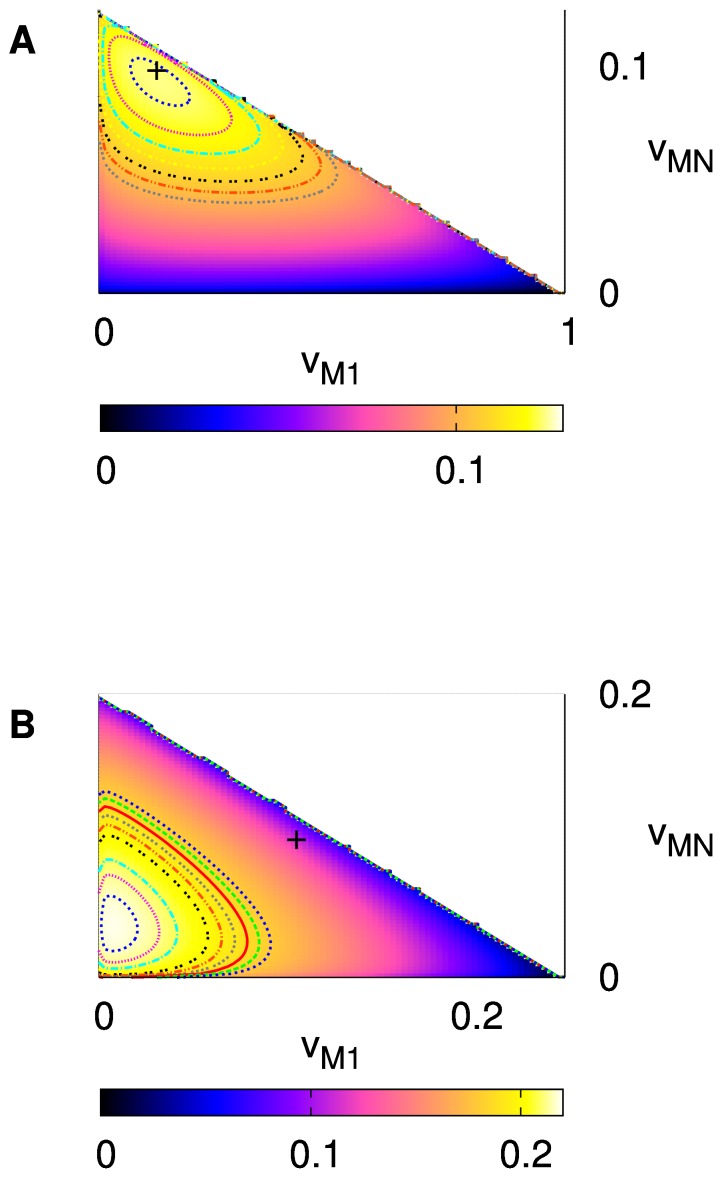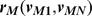 obtained by solving Eq. (8) within a region 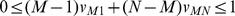 for 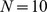. (A) 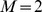. (B) 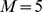. The crosses show 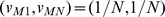.

In order to see whether egalitarians can eventually take over the society, we need to check whether the egalitarian preference remains as an attractive alternative when the society has both egalitarians and non-egalitarians with significant numbers. Let us imagine an inhomogeneous society where there are roughly two large groups: every person in one group of size 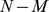 occupies a high index 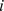 and believes that the welfare should be proportional to 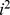. On the other hand, every person in the other group of size 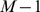 has a low index and an egalitarian preference. Our question is what kind of preference is good for a person on the border, i.e., with index 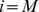. Again, since people with identical preferences will get the same amount of welfare, the focal person on the border need not distinguish the members in each group: suppose that she wishes 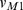 for each member in the egalitarian group and 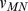 for each member in the non-egalitarian group. The normalization condition then determines her self-interest 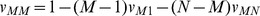. Depending on how she decides 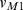 and 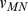, her final welfare 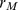 will be calculated by analyzing the following 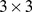 matrix,
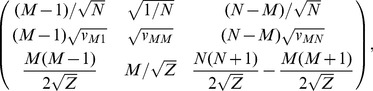
(8)where the second row describes this focal person 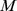. The conservation of the total welfare is imposed by setting 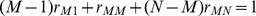. When 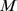 is small, the maximum of 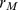 is close to the egalitarian solution 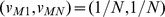 (Fig. 3A). It agrees with the result of the homogeneously unequal preferences given above since the egalitarian preference is still an absolute minority. Hence, if this person 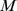 can choose her own preference, the society will possibly have one more egalitarian. As 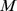 becomes larger, however, the situation gets different in that the maximum is located far from the egalitarian solution (Fig. 3B). It implies that the transition process toward the egalitarian direction may exhibit transient behavior, instead of being smooth all the time.

## Discussion

The theory of welfare is not an empty ideal as claimed in [7]: dealing with a society where everyone has an identical non-egalitarian preference, we have found that the theory recommends something very similar to an egalitarian preference, instead of just rubber-stamping the dominant non-egalitarian opinion. In addition, this finding shows that the egalitarian society is in fact the *only* strict Nash equilibrium. We therefore conclude that our analysis gives a strong support to equality of welfare by specifying which social and political conditions make it possible.

On the other hand, our conclusion implies that a society can encourage egalitarianism by guaranteeing freedom of communication so that everyone can constantly express her fulfillment in public. In this respect, we can perhaps mention one of the central messages in [7] that “liberty is essential to any process in which equality is defined and secured.” In particular, we would like to put an extra emphasis on the communicative aspect of the liberty.

On a longer perspective, our results suggest an explanation of how the concept of fairness could develop at a certain moment in the history of evolution when human beings became able to construct internal expectation for the future and understand others’ minds by communication. It is also worth stressing that our conclusion on egalitarianism as a strict Nash equilibrium under certain well-defined conditions is strong enough to open further theoretical extensions and empirical tests.

## Methods

We solve the eigenvalue problem of matrix 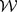 in each case analytically or numerically by using the power method.

## Supporting Information

Text S1 Details of the derivation of the Bhattacharyya measure.
(PDF)
