# Supplementary material for: Dworkin’s Paradox
Source: PLoS One. 2012 Jun 26;7(6):e38529. doi: 10.1371/journal.pone.0038529 (PMC3383739; doi:10.1371/journal.pone.0038529)
Supplement: Text S1 — Details of the derivation of the Bhattacharyya measure. (PDF) [file pone.0038529.s001.pdf]

Dworkin's Paradox

Seung Ki Baek[1], Jung-Kyoo Choi[2], Beom Jun Kim[3,*]
1 Integrated Science Laboratory, Umeå University, S-901 87 Umeå, Sweden (Present address: School of Physics, Korea Institute for Advanced Study, Seoul 130-722, Korea)
2 School of Economics and Trade, Kyungpook National University, Daegu 702-701, Korea
3 BK21 Physics Research Division and Department of Physics, Sungkyunkwan University, Suwon 440-746, Korea
∗ E-mail: beomjun@skku.edu

Derivation of the affinity function

Let us consider two arbitrary distributions, $\boldsymbol{p} = (p_1, \ldots, p_N)$ and $\boldsymbol{q} = (q_1, \ldots, q_N)$, with $p_i \geq 0$, $q_i \geq 0$, and $\sum_i^N p_i = \sum_i^N q_i = 1$. We will define an affinity function $\rho_N(\boldsymbol{p}, \boldsymbol{q})$ between them, whose specific functional form will be characterized by requiring the following postulates.

- P1: Separability

$$\rho_N \begin{pmatrix} p_1, \ldots, p_N \\ q_1, \ldots, q_N \end{pmatrix} = \rho_{N-k+1} \begin{pmatrix} P_k, p_{k+1}, \ldots, p_N \\ Q_k, q_{k+1}, \ldots, q_N \end{pmatrix}$$
$$+\Delta(P_k, Q_k) \left[\rho_k \begin{pmatrix} \frac{p_1}{P_k}, \ldots, \frac{p_k}{P_k} \\ \frac{q_1}{Q_k}, \ldots, \frac{q_k}{Q_k} \end{pmatrix} - 1 \right],$$

  where $P_k \equiv p_1 + p_2 + \cdots + p_k$ and $Q_k \equiv q_1 + q_2 + \cdots + q_k$ are positive with $1 < k < N$. In addition, $\Delta(P_k, Q_k)$ is a non-negative differentiable function and converges to zero as $P_k \to 0$ or $Q_k \to 0$. The situation can be described as follows: we first observe $\mathbf{p}' = (P_k, p_{k+1}, \ldots, p_N)$ and $\mathbf{q}' = (Q_k, q_{k+1}, \ldots, q_N)$, instead of the real $\mathbf{p}$ and $\mathbf{q}$. We can calculate affinity ρ_{N-k+1} in this resolution. The question is how affinity will change when we come to know the real $\mathbf{p}$ and $\mathbf{q}$. If the substructures inside P_k and Q_k have an exact affinity by $\rho_k = 1$, nothing will change from the previous calculation, i.e., $\rho_N = \rho_{N-k+1}$. If they actually had no affinity by $\rho_k = 0$ in this better resolution, on the other hand, the overall affinity should decrease by a certain amount Δ, which will be a function of P_k and Q_k. For example, for $P_k \ll 1$ or $Q_k \ll 1$, the decrement in ρ_N will be also vanishingly small even if the subpopulations inside them look completely different.

- P2: Invariance under permutation

$$\rho_3 \begin{pmatrix} p_1, p_2, p_3 \\ q_1, q_2, q_3 \end{pmatrix} = \rho_3 \begin{pmatrix} p_2, p_1, p_3 \\ q_2, q_1, q_3 \end{pmatrix} = \ldots = \rho_3 \begin{pmatrix} p_3, p_2, p_1 \\ q_3, q_2, q_1 \end{pmatrix}.$$

  Note that it is for $N = 3$, which is enough to prove the same property for general N in combination with the other postulates.

- P3: Non-negativity

$$\rho_N(\boldsymbol{p}, \boldsymbol{q}) \geq 0,$$

  where $\rho_N(\boldsymbol{p}, \boldsymbol{q}) = 0$ if and only if $\boldsymbol{p}$ is orthogonal to $\boldsymbol{q}$, whereas a maximum value is obtained if and only if $\boldsymbol{p} = \boldsymbol{q}$.

- P4: Symmetry

$$\rho_N(\boldsymbol{p}, \boldsymbol{q}) = \rho_N(\boldsymbol{q}, \boldsymbol{p}).$$

Let us explain some direct consequences of P1. For $N=2$, it yields a trivial equality. For $N=3$, we have

$$\rho_3\begin{pmatrix}p_1,p_2,p_3\\q_1,q_2,q_3\end{pmatrix}=\rho_2\begin{pmatrix}P_2,p_3\\Q_2,q_3\end{pmatrix}+\Delta(P_2,Q_2)\left[\rho_2\begin{pmatrix}\frac{p_1}{P_2},\frac{p_2}{P_2}\\\frac{q_1}{Q_2},\frac{q_2}{Q_2}\end{pmatrix}-1\right].$$

For $N=4$, we will see two different ways to calculate affinity between (P_3,p_4) and (Q_3,q_4). The first way is the following:

$$\begin{aligned}&\rho_4\begin{pmatrix}p_1,\ldots,p_4\\q_1,\ldots,q_4\end{pmatrix}\\=\;&\rho_2\begin{pmatrix}P_3,p_4\\Q_3,q_4\end{pmatrix}+\Delta(P_3,Q_3)\left[\rho_3\begin{pmatrix}\frac{p_1}{P_3},\frac{p_2}{P_3},\frac{p_3}{P_3}\\\frac{q_1}{Q_3},\frac{q_2}{Q_3},\frac{q_3}{Q_3}\end{pmatrix}-1\right]\\=\;&\rho_2\begin{pmatrix}P_3,p_4\\Q_3,q_4\end{pmatrix}+\Delta(P_3,Q_3)\left\{\rho_2\begin{pmatrix}\frac{P_2}{P_3},\frac{p_3}{P_3}\\\frac{Q_2}{Q_3},\frac{q_3}{Q_3}\end{pmatrix}\right.\\&\left.+\Delta\left(\frac{P_2}{P_3},\frac{Q_2}{Q_3}\right)\left[\rho_2\begin{pmatrix}\frac{p_1}{P_2},\frac{p_2}{P_2}\\\frac{q_1}{Q_2},\frac{q_2}{Q_2}\end{pmatrix}-1\right]-1\right\}.\end{aligned}\tag{1}$$

We then consider another way to calculate the same quantity as follows:

$$\begin{aligned}&\rho_4\begin{pmatrix}p_1,\ldots,p_4\\q_1,\ldots,q_4\end{pmatrix}\\=\;&\rho_3\begin{pmatrix}P_2,p_3,p_4\\Q_2,q_3,q_4\end{pmatrix}+\Delta(P_2,Q_2)\left[\rho_2\begin{pmatrix}\frac{p_1}{P_2},\frac{p_2}{P_2}\\\frac{q_1}{Q_2},\frac{q_2}{Q_2}\end{pmatrix}-1\right]\\=\;&\rho_2\begin{pmatrix}P_3,p_4\\Q_3,q_4\end{pmatrix}+\Delta(P_3,Q_3)\left[\rho_2\begin{pmatrix}\frac{P_2}{P_3},\frac{p_3}{P_3}\\\frac{Q_2}{Q_3},\frac{q_3}{Q_3}\end{pmatrix}-1\right]\\&+\Delta(P_2,Q_2)\left[\rho_2\begin{pmatrix}\frac{p_1}{P_2},\frac{p_2}{P_2}\\\frac{q_1}{Q_2},\frac{q_2}{Q_2}\end{pmatrix}-1\right].\end{aligned}\tag{2}$$

Comparing Eq. (1) with Eq. (2), we see that

$$\Delta(P_3,Q_3)\Delta\left(\frac{P_2}{P_3},\frac{Q_2}{Q_3}\right)=\Delta(P_2,Q_2).\tag{3}$$

Note that P_2/P_3 can take any value between zero and one, regardless of P_3, and that Q_2/Q_3 is also independent of Q_3 in the same way, as long as P_3 and Q_3 are nonzero.

Now we define

$$g(x,y)\equiv\rho_2\begin{pmatrix}x,1-x\\y,1-y\end{pmatrix}-1,\tag{4}$$

for $x,y\in[0,1]$. From P1 and P2, it is straightforward to verify

$$g(x,y)=g(1-x,1-y),\tag{5}$$

since

$$\begin{aligned}&\rho_3\begin{pmatrix}p_1,p_2,p_3\\q_1,q_2,q_3\end{pmatrix}=\rho_3\begin{pmatrix}p_2,p_1,p_3\\q_2,q_1,q_3\end{pmatrix}\\=\;&\rho_2\begin{pmatrix}P_2,p_3\\Q_2,q_3\end{pmatrix}+\Delta(P_2,Q_2)\left[\rho_2\begin{pmatrix}\frac{p_1}{P_2},\frac{p_2}{P_2}\\\frac{q_1}{Q_2},\frac{q_2}{Q_2}\end{pmatrix}-1\right]\\=\;&\rho_2\begin{pmatrix}P_2,p_3\\Q_2,q_3\end{pmatrix}+\Delta(P_2,Q_2)\left[\rho_2\begin{pmatrix}\frac{p_2}{P_2},\frac{p_1}{P_2}\\\frac{q_2}{Q_2},\frac{q_1}{Q_2}\end{pmatrix}-1\right].\end{aligned}$$

Applying P1 to another permutation

$$\rho_3\begin{pmatrix}p_1,p_2,p_3\\q_1,q_2,q_3\end{pmatrix}=\rho_3\begin{pmatrix}p_3,p_2,p_1\\q_3,q_2,q_1\end{pmatrix},$$

we are directly led to

$$\begin{aligned}&g(p_3,q_3)+\Delta(1-p_3,1-q_3)g\left(\frac{p_1}{1-p_3},\frac{q_1}{1-q_3}\right)\\=\;&g(p_1,q_1)+\Delta(1-p_1,1-q_1)g\left(\frac{p_3}{1-p_1},\frac{q_3}{1-q_1}\right),\end{aligned}$$

where $p_1,p_3,q_1,q_3\in[0,1)$. Let us redefine the variables as $\frac{p_1}{1-p_3}\equiv p$, $\frac{q_1}{1-q_3}\equiv q$, $1-p_3\equiv r$, and $1-q_3\equiv s$. Each of these new variables p, q, r, and s can take an arbitrary value independently of one another, as long as $p,q\in[0,1]$ and $r,s\in(0,1)$. The above equality can be then rewritten as

$$\begin{aligned}&g(r,s)+\Delta(r,s)g(p,q)\\&=g(pr,qs)+\Delta(1-pr,1-qs)g\left(\tfrac{1-r}{1-pr},\tfrac{1-s}{1-qs}\right).\end{aligned}\tag{6}$$

Defining a function

$$f(p,q,r,s)\equiv g(r,s)+\left[\Delta(r,s)+\Delta(1-r,1-s)\right]g(p,q)\tag{7}$$

for $p,q,r,s\in(0,1)$, we apply Eq. (6) to $f(p,q,r,s)$ to obtain

$$\begin{aligned}f(p,q,r,s)\;=\;&g(pr,qs)\\&+\Delta(1-pr,1-qs)g\left(\frac{1-r}{1-pr},\frac{1-s}{1-qs}\right)\\&+\Delta(1-r,1-s)g(p,q).\end{aligned}$$

Note from Eq. (3) that

$$\begin{aligned}&\Delta(p_2+p_3,q_2+q_3)\Delta\left(\frac{p_3}{p_2+p_3},\frac{q_3}{q_2+q_3}\right)\\=\;&\Delta(1-pr,1-qs)\Delta\left(\frac{1-r}{1-pr},\frac{1-s}{1-qs}\right)\\=\;&\Delta(1-r,1-s).\end{aligned}$$

Hence, we see that

$$\begin{aligned}&f(p,q,r,s)\\=\;&g(pr,qs)+\Delta(1-pr,1-qs)g\left(\frac{1-r}{1-pr},\frac{1-s}{1-qs}\right)\\&+\Delta(1-pr,1-qs)\Delta\left(\frac{1-r}{1-pr},\frac{1-s}{1-qs}\right)g(p,q)\\=\;&g(pr,qs)+\Delta(1-pr,1-qs)\left[g\left(\frac{1-r}{1-pr},\frac{1-s}{1-qs}\right)\right.\\&\left.+\Delta\left(\frac{1-r}{1-pr},\frac{1-s}{1-qs}\right)g(p,q)\right]\qquad(8)\\=\;&g(pr,qs)+\Delta(1-pr,1-qs)\left\{g\left[\frac{p(1-r)}{1-pr},\frac{q(1-s)}{1-qs}\right]\right.\\&\left.+\,\Delta\left(\frac{1-p}{1-qr},\frac{1-q}{1-qs}\right)g(r,s)\right\},\qquad(9)\end{aligned}$$

where the last equality comes from Eq. (6). Equation (5) then gives us

$$g\left[\frac{p(1-r)}{1-pr}, \frac{q(1-s)}{1-qs}\right] = g\left(\frac{1-p}{1-pr}, \frac{1-q}{1-qs}\right),$$

relating Eqs. (8) and (9) as follows:

$$\begin{aligned}
& f(p,q,r,s) \\
= \; & g(pr,qs) + \Delta(1-pr, 1-qs)\left[g\left(\frac{1-r}{1-pr}, \frac{1-s}{1-qs}\right)\right. \\
& \left. + \, \Delta\left(\frac{1-r}{1-pr}, \frac{1-s}{1-qs}\right) g(p,q)\right] \\
= \; & g(pr,qs) + \Delta(1-pr, 1-qs)\left[g\left(\frac{1-p}{1-pr}, \frac{1-q}{1-qs}\right)\right. \\
& \left. + \, \Delta\left(\frac{1-p}{1-pr}, \frac{1-q}{1-qs}\right) g(r,s)\right].
\end{aligned}$$

In short, we have just confirmed that

$$f(p,q,r,s) = f(r,s,p,q).$$

From the definition of $f(p,q,r,s)$ in Eq. (7), we see that

$$\frac{g(r,s)}{g(p,q)} = \frac{\Delta(r,s) + \Delta(1-r,1-s) - 1}{\Delta(p,q) + \Delta(1-p,1-q) - 1}.$$

For this to be true for every independent set of (p,q,r,s), it must be that

$$g(r,s) = C[\Delta(r,s) + \Delta(1-r,1-s) - 1]$$

with a certain constant C. Then P1 says that

$$\lim_{\substack{r\to 0 \\ s\to 1}} g(r,s) = -C,$$

which is the lowest possible value of $g(r,s)$. However, according to the definition of $g(r,s)$ in Eq. (4) and P3, this lower bound should be -1. In other words, $C = 1$ and

$$\rho_2 \begin{pmatrix} r, 1-r \\ s, 1-s \end{pmatrix} = \Delta(r,s) + \Delta(1-r,1-s).$$

We have restricted our variables as $r \in (0,1)$ and $s \in (0,1)$, but it is not difficult to check that the above expression can be readily used in $r \in [0,1]$ and $s \in [0,1]$ when Δ is characterized as will be discussed below. Then P1 works as a recursive relation, yielding an expression for general N:

$$\rho_N = \sum_{i=1}^{N} \Delta(p_i, q_i). \tag{10}$$

Now we ask ourselves how Δ should look. P1 and P4 imply that Δ can be expanded as $\Delta(p_i, q_i) = c_1(p_i q_i)^{\alpha_1} + c_2(p_i q_i)^{\alpha_2} + \cdots$ with coefficients c_n and exponents $\alpha_n > 0$. So let us write

$$\rho_N = \sum_{i=1}^{N} \sum_{n=1}^{\infty} c_n (p_i q_i)^{\alpha_n}.$$

If we define $l_\alpha(\boldsymbol{p},\boldsymbol{q}) \equiv \sum_{i=1}^N (p_i q_i)^\alpha$, it means that ρ_N should be of the following form,

$$\rho_N(\boldsymbol{p},\boldsymbol{q}) = \sum_{n=1}^{\infty} c_n l_{\alpha_n}(\boldsymbol{p},\boldsymbol{q}). \tag{11}$$

Among every possible l_α, it is only $l_{1/2}$ that satisfies the maximization condition in P3. This can be shown by variational calculus using a Lagrange multiplier μ,

$$\frac{\partial}{\partial q_i}\left[l_\alpha(\boldsymbol{p},\boldsymbol{q}) - \mu \sum_{j=1}^N q_j \right]_{q_i=p_i} = \alpha p_i^{2\alpha-1} - \mu = 0,$$

which is satisfied for any p_i when $\alpha = 1/2$ and $\mu = \alpha$. Hence we separate this term from the others in Eq. (11) as

$$\rho_N(\boldsymbol{p},\boldsymbol{q}) = c l_{1/2}(\boldsymbol{p},\boldsymbol{q}) + {\sum_n}' c_n l_{\alpha_n}(\boldsymbol{p},\boldsymbol{q}),$$

where $\sum'$ means that $\alpha_n = 1/2$ is excluded from the summation. There exists a maximum value in ρ_N, which is obtained by

$$\begin{aligned} \rho_N(\boldsymbol{p},\boldsymbol{p}) &= c l_{1/2}(\boldsymbol{p},\boldsymbol{p}) + {\sum_n}' c_n l_{\alpha_n}(\boldsymbol{p},\boldsymbol{p}) \\ &= c + {\sum_n}' c_n \sum_i p_i^{2\alpha_n}, \end{aligned}$$

noting that $l_{1/2}(\boldsymbol{p},\boldsymbol{p}) = \sum_i p_i = 1$. Since this value is the same for every $\boldsymbol{p}$, the last term should be kept constant for any p_i, and we have to choose $c_n = 0$ for every n in the second term. To sum up, our affinity function is characterized as

$$\rho_N(\boldsymbol{p},\boldsymbol{q}) = c \sum_{i=1}^N (p_i q_i)^{1/2}$$

with a positive constant c, the maximum value of ρ_N.
